# Supplementary material for: NAMPT and PARylation Are Involved in the Pathogenesis of Atopic Dermatitis
Source: Int J Mol Sci. 2023 Apr 28;24(9):7992. doi: 10.3390/ijms24097992 (PMC10178103; doi:10.3390/ijms24097992)
Supplement: Supplementary file 1 [file ijms-24-07992-s001.zip › ijms-2229164-supplementary.pdf]

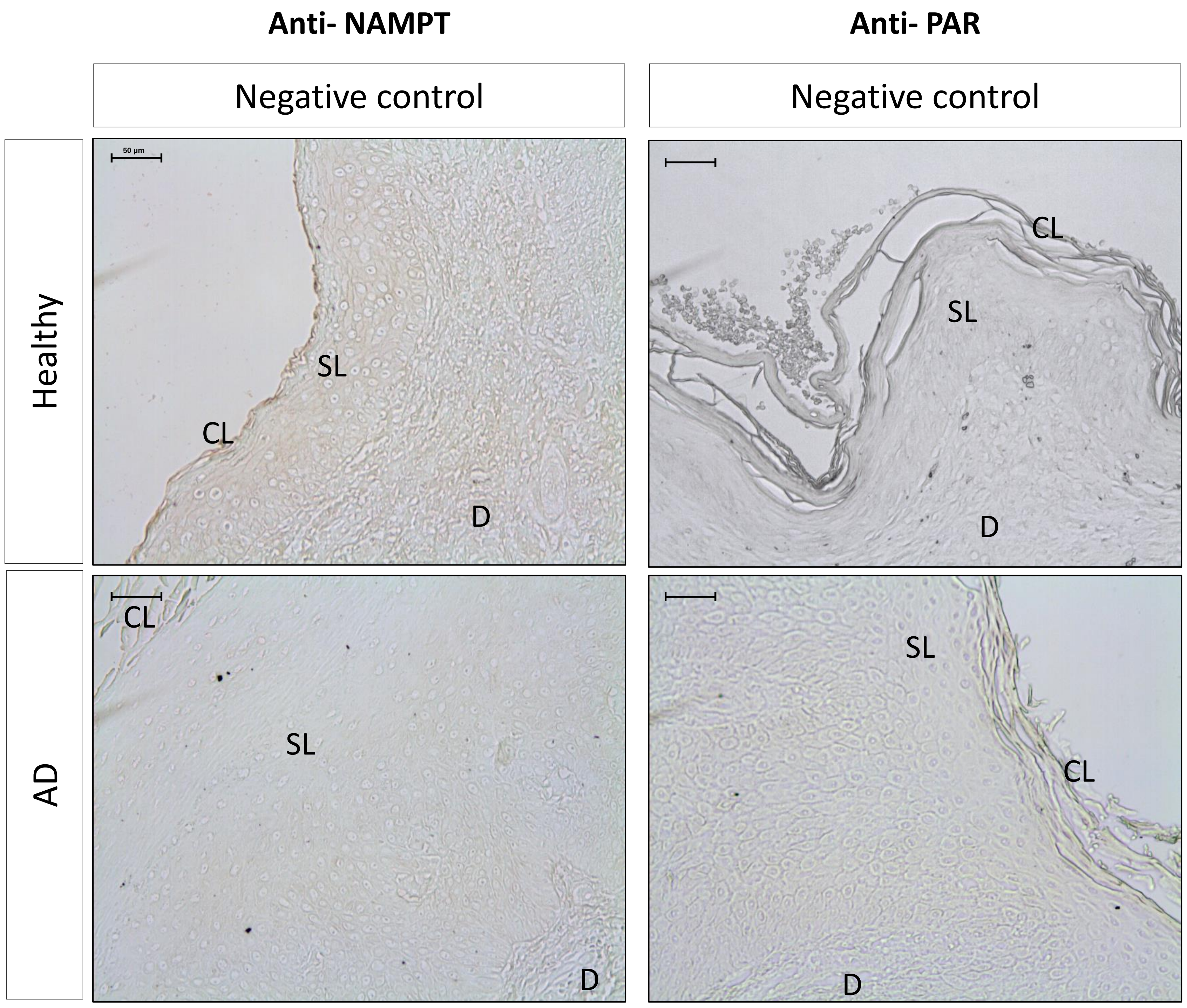

Supplemental Figure S1 (related to Figure 1). Negative control of NAMPT and PAR immunostaining in skin biopsies of healthy and AD patients. Representative images of biopsy sections from healthy and AD skin biopsies that have been immunostained by omitting the primary antibodies. Scale bar is 50 μm in all panels. CL: cornified layer; D: dermis; S: spinous layer.

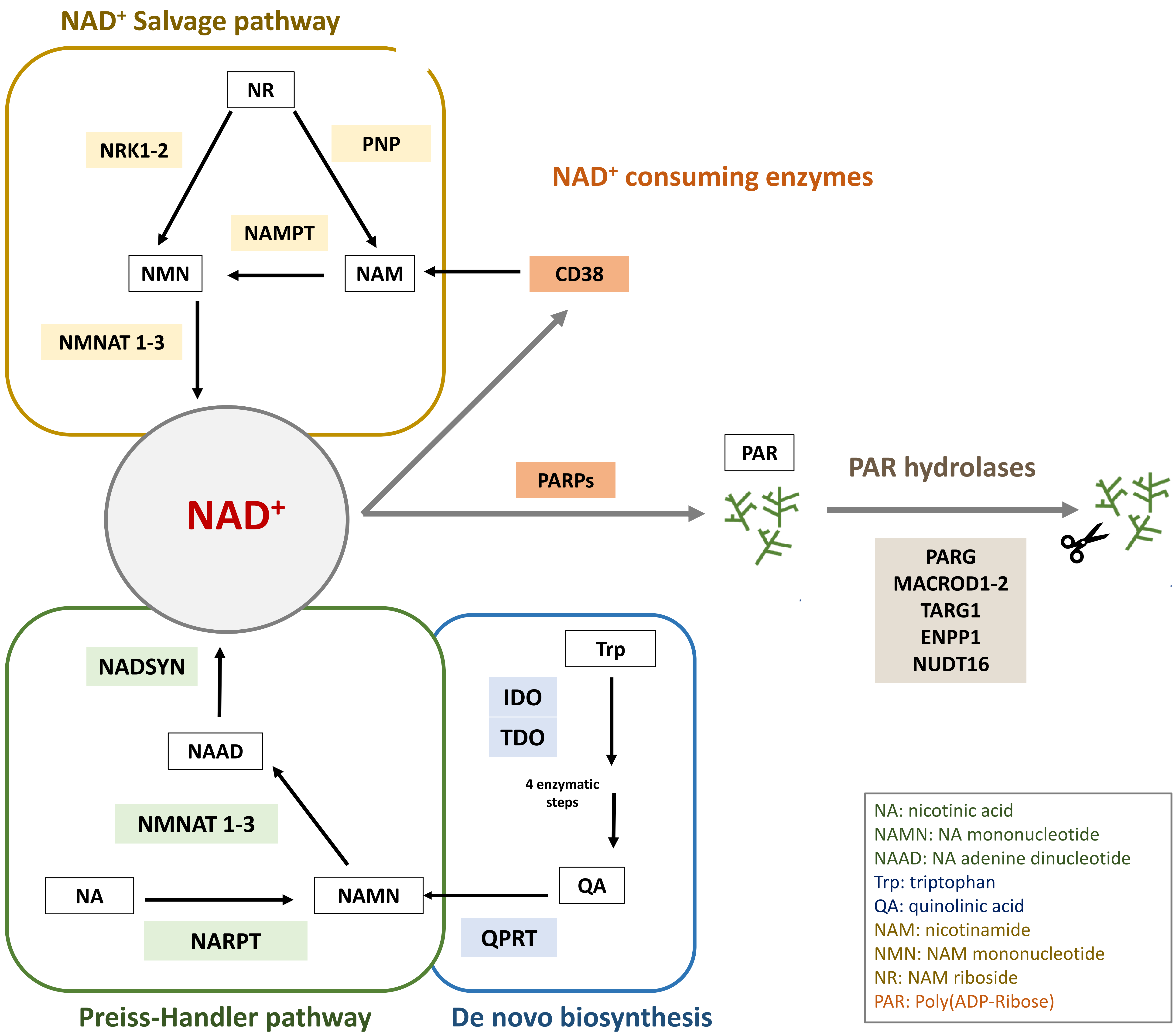

Supplemental Figure S2 (related to Figures 2 and 3). Schemes showing NAD$^+$ and PAR metabolic pathways.

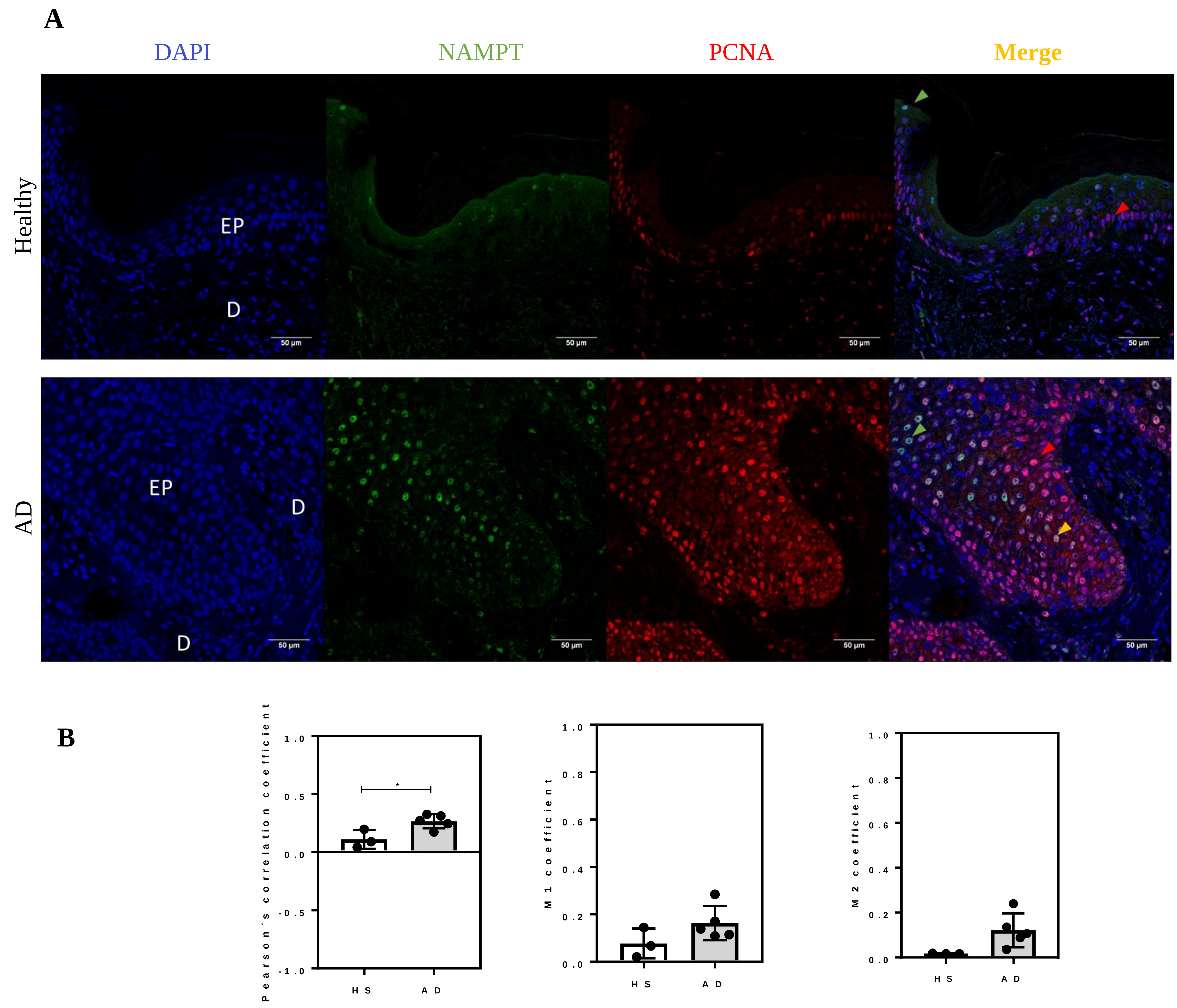

Supplemental Figure S3. Double immunofluorescence analysis of NAMPT and PCNA in skin human biopsies from healthy donors and patients of atopic dermatitis. (A) Representative images of skin biopsies from healthy subjects (HS) and AD immunostained with rabbit anti-NAMPT (1:1000, green) and mouse monoclonal anti-PCNA (1:1000, red) antibodies, and then counterstained with DAPI. Sections were examined under a confocal microscope (Leica STELLARIS 8) and further processed with Leica software and ImageJ. Cornified layer; SL, Spinous layer; D, dermis. (B) Quantification of the colocalization in images from healthy donors (n=3) and AD patients (n=5). The correlation of NAPMT and PCNA fluorescence intensity was determined using ImageJ and the JACoP plugin (Lachmanovich method), and specifically the Pearson correlation coefficient and Manders (M1 and M2) coefficients (see Methods and Materials Section). $p \leq 0.05$.

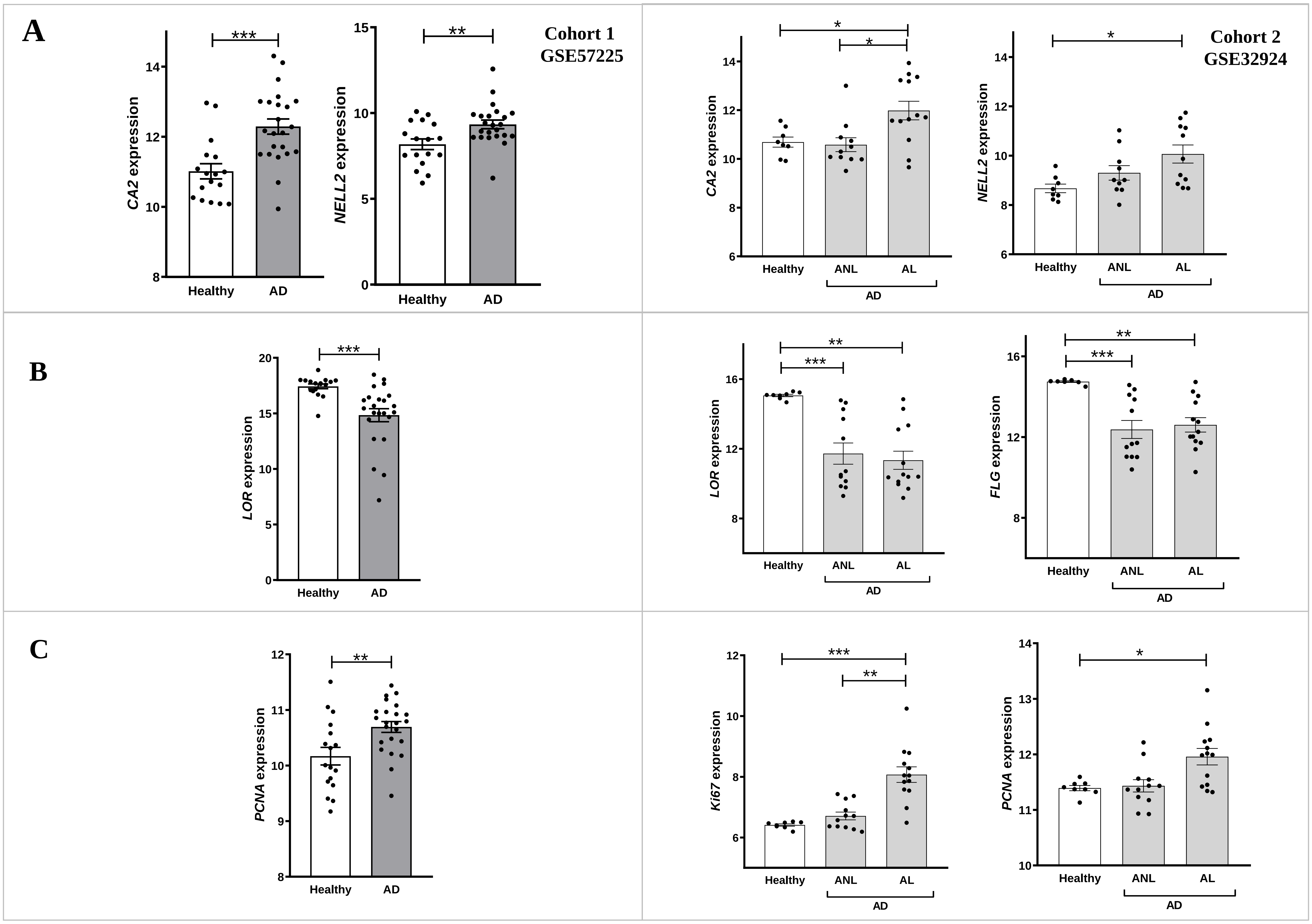

Supplemental Figure S4 (related to Figure 5). Differential expression profiles of inflammation, epidermal differentiation and proliferation biomarkers involved in AD. Transcriptomic data of inflammatory (A), epidermal differentiation, (B) and proliferation (C) markers of AD obtained from two different human GEO database cohorts, GSE57225 and GSE32924. In cohort 1, AD was compared with healthy skin samples (A-B) and in cohort 2 skin with lesional (AL) and non-lesional (ANL) AD was compared with healthy skin samples. Each dot represents one individual and the mean ± SEM of each group is also shown. P values were calculated by unpaired Student’s t test and 1-way ANOVA and Tukey's multiple range test, as appropriate. *p ≤ 0.05, **p ≤ 0.01, ***p ≤ 0.001.

Supplemental Table S1. Sequences of primers used for RT-qPCR

Gene	Name	Forward (5'-3')	Reverse (5'-3')
CA2	Carbonic anhydrase II	aacaatggtcatgctttcaacg	tgtccatcaagtgaacccag
NELL2	Neuron-specific Nel-like protein 2	gaaagggtagtagaaaagcc	acccttaaaatatccatgcg
CCL26	C-C motif chemokine ligand 26	aaaagaggcaagaaagtct	ccatgtagccttcagaaaag
FLG	Filaggrin	acttcactgagtttcttctgatggtatt	tccagacttgagggtctttttctg
LOR	Loricrin	aggttaagacatgaaggatttgcaa	ggcaccgatgggcttagag
PCNA	Proliferating Cell Nuclear Antigen	ctgtgtagtaaagatgccttc	tctctatggtaacagcttcc
NAMPT	Nicotinamide Phosphoribosyltransferase	ctaatggccttgggattaac	tccagtgtaacaaaattccc
ACTB	Actin Beta	ggcaccacaccttctacaatg	gtggtggtgaagctgtagcc
